# 

**Published:** 1899-08

**Authors:** 


### CONTENTS.

ORIGINAL ARTICLES:
Gangrenous Grease, or Dermatitis Gangrenosa. By R. C. MOORE, M.D.C. . . . 469
Dairy Sanitation. By C. M. DAY, D.V.M. 473
Meat-inspection and Milk-inspection. By Dr. H. F. PALMER . . . . . . 477
Antisepsis in Veterinary Practice. By H. G. PATTERSON, D.V.S. . . . . . 482
Valedictory Address. By CLYDE S. HESS. 486
Diseases of the Udder and Teats. By W. H. AUSTIN, V.S. . . . . . . . 488
Deadly Nightshade, or Belladonna. By G. C. KESLER, V.S. . . . . . . . 491
Eserine. By Dr. E. E. BITTLES . . . 492
SELECTIONS:
Rabies in England. By M. E. LECLAINCHE. Translated by MAZYCK P. RAVENEL, M.D. 494
Parasitic Disease of Rabbits. By VETERINARY SURGEON DESMOND . . . 497
Milk in Boston . . . . . . . . 500
THERAPEUTICAL AND PHARMACEUTICAL NOTES 501
DEPARTMENT OF SURGERY:
Clinical Diagnosis of Nasal Discharges . 507
REPORTS OF CASES:
Case of Rabies, Including Experiments on Rabbits. By E. M. RANCK, V.M.D. . 510
Epizootic Cellulitis Among Horses. By Dr. MANLEY. . . . . . . . . . 511
Destruction of Jugular Vein and Carotid Artery Following Injection of Barium Chloride. By Dr. GEORGE L. WARNER . 512
EDITORIAL:
A Fearful Responsibility—Our Profession's Loss—New York in September . . . 513
MARRIAGE—NECROLOGY . . . . . . 516
A. V. M. A. . . . . . . . . 518
AMONG THE COLLEGES . . . . . . 523
AMONG THE BOARDS OF EXAMINERS . . 524
NOTES . . . . . . . . . . 525
HOW THEY ADVERTISE IN THE NORTHWEST 527
SOCIETY PROCEEDINGS:
Westchester County Association—Maine—Missouri Valley — Chicago . . . 528
PERSONALS . . . . . . . . . 534

